# Control of antimicrobial resistance in Canada: any lessons to learn?

**DOI:** 10.1186/2047-2994-1-6

**Published:** 2012-02-02

**Authors:** Lindsay E Nicolle

**Affiliations:** 1 University of Manitoba, Health Sciences Centre, Room GG443 - 820 Sherbrook Street, Winnipeg, MB R3A 1R9, Canada

**Keywords:** antimicrobial resistance, Canada, antimicrobial stewardship

## Abstract

**Background:**

Over the past 15 years, repeated national meetings have developed recommendations for a Canadian antimicrobial resistance strategy. Despite this, in 2011 there is no comprehensive, integrated national program with appropriate governance and funding to address antimicrobial resistance.

**Findings:**

The Public Health Agency of Canada supports a reference laboratory for diagnosis and characterization of selected resistant strains, targeted surveillance programs which monitor resistance trends for selected animal and human organisms, development of national infection control guidelines including for antimicrobial resistant organisms, and a few local pilot projects to address community acquired MRSA. Sporadic programs of variable intensity and quality are supported by some provinces, health regions and individual facilities but these are not comprehensive, standardized or integrated. Individual researchers and research groups, however, have published substantial information describing the prevalence and impact of resistance in Canada.

**Conclusions:**

Current review of activities by the Public Health Agency of Canada and initiatives by the National Coordinating Centre for Infectious Diseases may move the country forward in developing an effective national approach to address antimicrobial resistance.

## Background

Over the past 15 years, the problem of antimicrobial resistance in Canada has been repeatedly discussed at national meetings, with recommendations developed to address this problem in the Canadian context. Some key documents from these meetings are:

1. Consensus Conference: Controlling antimicrobial resistance: An integrated action plan for Canadians (1997) [1]. http://www.phac-aspc.gc.ca/publications-eng.php#C

2. Uses of Antimicrobials in Food Animals in Canada: Impact on Resistance and Human Health. Report of the Advisory Committee on Resistance and Human Health (2002) [2]. http://www.hc-sc.gc.ca/dhp-mps/pubs/vet/amr-ram_final_report-rapport_06-27cp-pc-eng.php

3. Proposed National Action plan to Combat Antimicrobial Resistance. Canadian Committee on Antimicrobial Resistance (2004).

4. The Pan-Canadian Stakeholder Consultations on Antimicrobial Resistance, Canadian Committee on Antibiotic Resistance (2009).

5. Consultation: Community acquired antimicrobial resistance, National Collaborating Centre for Infectious Diseases (2010). http://www.nccid.ca

However, despite universal acknowledgment that antimicrobial resistance is an important Canadian problem together with consistent interest in addressing the issue at a national level, in 2011 there is no comprehensive, integrated, national Canadian program with appropriate governance and funding for limiting the progression of antimicrobial resistance.

## Activities

The Canadian Committee on Antibiotic Resistance was established after the 1997 Consensus Conference to co-ordinate and provided leadership for antimicrobial resistance initiatives in Canada. It functioned until 2009, spearheading a number of initiatives, when it was dissolved after funding was discontinued. The Public Health Agency of Canada currently supports a reference laboratory for diagnosis and characterization of resistant bacterial strains, as well as targeted surveillance programs to monitor resistance trends for selected animal and human pathogens, development of national infection control guidelines including for management of antimicrobial resistant organisms in health care facilities, and some pilot projects to address community acquired MRSA. Programs of variable intensity and quality are also supported by some individual provinces, health regions and health care facilities, but these are not comprehensive, standardized or integrated. Individual researchers and research groups have published substantial information which describes the prevalence and impact of antimicrobial resistance in Canada. These studies consistently report that the prevalence of antimicrobial resistance in bacterial isolates from Canada tends to be lower than reported for the United States, but that trends over time, with few exceptions (fluoroquinolone resistant *S. pneumoniae*), are to increasing resistance.

## Successes

While there has been limited progress towards a comprehensive national program, some unique programs to address selected aspects of antimicrobial resistance have been developed and maintained. These programs include:

1. "Do Bugs need Drugs" http://www.dodrugsneedbugs.org

A community based public and professional education program promoting handwashing and responsible use of antibiotics to address the problem of antimicrobial resistance. Programs are available for physicians ("Bugs and Drugs" Antimicrobial Indications Handbook), pharmacists, nurses, occupational health nurses, teachers, schools, daycare centres, assisted living centres, parents, children and the public. This program was initially developed with government support in the province of Alberta, and subsequently introduced into a second province, British Columbia. Selected program components are also used in some other jurisdictions in Canada. Evaluation of the impact of the program or individual program components on community antimicrobial resistance has not, however, been reported.

2. Northern Antibiotic Resistance Partnership http://www.Germsaway.ca

This community, federal government, and academic partnership has used a public health approach to develop guidelines and teaching materials for prevention and management of CA-MRSA infections in isolated northern communities, many of which have a very high incidence of infections with these organisms. The program includes an interactive video game for schools, an educational tool, handwashing posters aimed at children, slide presentations, education podcasts, radio broadcasts in English and local Aboriginal languages, and CA-MRSA pamphlets for physicians offices and health centres in the relevant communities. Following the implementation of the program, a decrease in CA-MRSA infection in some of the program communities has been observed, but the extent to which this can be attributed to the program activities is not determined.

3. Canadian Nosocomial Infection Surveillance Program (CNISP) http://www.phac-aspc.gc.ca/nois-sinp/survprog-eng.php

This partnership between AMMI-Canada (Association of Medical Microbiology and Infectious Diseases - Canada) and the Public Health Agency of Canada was initially established in 1994. It currently supports focused monitoring of healthcare acquired infections, including selected antimicrobial resistant organisms (including MRSA [3], VRE [4], *C. difficile*, resistant gram negatives), at 37 sentinal hospitals - most of the Canadian tertiary care centres. Specific projects have described the prevalence, incidence, trends over time, and some impacts of antimicrobial resistant organisms in the Canadian Health Care system.

4. Canadian Integrated Program of Antimicrobial Resistance Surveillance (CIPARS) http://www.phac-aspc.gc.ca/cipars-picra/index-eng.php

This program monitors trends in antimicrobial use and resistance for selected food borne bacterial organisms (*E. coli 0157:H7*, Salmonella spp, Campylobacter spp) isolated from humans, agricultural animals, and food sources across Canada. Surveillance includes results of cultures collected from abattoirs, animal clinical specimens, animal feed, human clinical specimens, on-farm, and retail, as well as of human and animal antimicrobial use. There are annual reports for 2002 - 2007, preliminary reports for 2008, 2009, and agrifood quarterly summaries of Salmonella spp (most recent Oct - Dec 2010).

## Conclusion

Despite some successes with these and other isolated activities, it is acknowledged that an integrated, national antimicrobial resistance policy and program incorporating an effective governance structure and appropriate sustained funding is essential to modify the progression of antimicrobial resistance in Canada and limit potential negative effects associated with resistance. This must include a comprehensive national antimicrobial resistance surveillance system and coherent definitions and programs for antimicrobial stewardship in health care facilities, the community, animal and environmental settings. Some current activities, it is hoped, may move this agenda forward. The Public Health Agency of Canada has recently reviewed the status of antimicrobial resistance activities within the organization and across Canada and identified gaps in antimicrobial resistance activities at the federal agency level. These have been summarized in the context of surveillance, research, best practices for limitation and control of resistance, and governance policy and legislation. The agency has said it will commit resources internally to develop a specific focus addressing antimicrobial resistance as a response to this review. The National Collaborating Centre on Infectious Diseases has identified community antimicrobial resistance as a Centre priority. It spearheaded a discussion of needs and process to address community antimicrobial resistance, and also recently issued a request for applications to critically review current activities addressing antimicrobial resistance surveillance across Canada, together with identification of needs to move forward in developing a relevant, effective national surveillance program. These initiatives may be helpful, if sustained, to promote the development of a national antimicrobial resistance program.

## Competing interests

The author declares that they have no competing interests.

## Authors' contributions

LEN is fully responsible for data acquisition, analysis, interpretation and writing of the manuscript.
